# Another addition to the unsolved puzzle of DCI

**DOI:** 10.1007/s00701-022-05400-w

**Published:** 2022-11-09

**Authors:** Joachim Oertel, Fritz Teping

**Affiliations:** Klinik für Neurochirurgie, Universitaet des Saarlandes, Medizinische Fakultaet, Gebaeude 90.5, 66421 Homburg/Saar, Germany

Aneurysmal subarachnoid haemorrhage and related morbidities remain one of the most complex conditions in neurosurgery. Overcoming the attribution of unfavourable outcomes solely to primary brain damage and angiographic vasospasm, many studies have contributed to the identification of numerous processes and events leading to poor outcomes after aSAH. Since the early definition of delayed cerebral ischemia (DCI) [1, 2], it has been one of the most important outcome parameters in studies focused on aSAH. However, the more aspects are investigated, the more complex its pathophysiology appears to be. In order to keep track of relevant findings, well-structured and up-to-date reviews are an important tool to bundle and extract relevant information.

Rehman et al. present such a review on the predictive value of sex for delayed cerebral ischemia (DCI) and hydrocephalus after aSAH. As pointed out by the authors, available results on the influence of sex concerning DCI and malresorptive hydrocephalus in particular have been inconsistent in the past. The presented meta-analysis was able to statistically confirm the common perception of female patients being more likely to develop DCI after aSAH. However, no such correlation could be identified for shunt-dependant posthaemorrhagic hydrocephalus. Whilst the presented results are reasonable and suggest a higher alertness when treating female patients with aSAH, the underlying pathophysiology remains too elusive to change current clinical management. It should therefore be addressed in upcoming studies considering gender-specific criteria, such as hormones or genetic features, in particular.

However, one of the major general problems of aSAH studies can be observed here: Delayed cerebral ischemia is still defined very heterogeneously throughout current literature. Considering the particular subject of gender influence, the authors were able to meticulously identify congruent definitions, even though they have been named differently in the various publications. However, homogeneous use of DCI definition would clearly simplify comparing results from different studies to extract general conclusions. The importance of clear definitions not only for outcome parameters, but also for the initial classification of SAH was recently emphasised again [3]. Thus, united efforts should be put into standardisation of scientific criteria and definitions in order to approach the still unsolved puzzle of morbidity after aSAH even more efficiently.
